# Enhancing the role of the social network in activity (re)engagement post-stroke: a focus group study with rehabilitation professionals

**DOI:** 10.1186/s12875-022-01897-3

**Published:** 2022-11-17

**Authors:** Dinja J. van der Veen, Sandra Jellema, Philip J. van der Wees, Maud J. L. Graff, Bert J. M. de Swart, Esther M. J. Steultjens

**Affiliations:** 1 Radboud University Medical Center, Radboud Institute for Health Sciences, IQ Healthcare, Nijmegen, The Netherlands; 2 HAN University of Applied Sciences, Nijmegen, The Netherlands; 3 Department of Rehabilitation, Radboud University Medical Center, Donders Institute for Brain, Cognition and Behaviour, Nijmegen, The Netherlands

**Keywords:** Stroke, Rehabilitation, Informal Caregiving, Social Network, Valued Activities, Interprofessional Collaboration, Implementation Strategies

## Abstract

**Background:**

People post-stroke are at risk of not being able to participate in valued activities. It is important that rehabilitation professionals prepare people post-stroke for the transition home and provide needed support when they live at home. Several authors have suggested that members of the broad social network should play an active role in rehabilitation. This includes informing them about the importance of activity (re)engagement post-stroke and learning strategies to provide support. It is not clear when and how the broad social network can best be equipped to provide adequate activity support. This study aimed to explore stroke professionals’ perspectives on strategies that establish a social network that supports activity (re)engagement of people post-stroke, when strategies are best implemented, and the factors that influence the implementation of these strategies.

**Methods:**

Two focus groups were executed. Content analysis was used to analyze the transcripts of the recorded conversations.

**Results:**

Eighteen professionals with various professional backgrounds and roles in treating people post-stroke participated. Strategies to establish a supportive social network included identifying, expanding, informing, and actively engaging network members. Working with the network in the immediate post-stroke phase was regarded as important for improving long-term activity outcomes. Participants expressed that most strategies to equip the social network to support people post-stroke need to take place within community care. However, the participants experienced difficulties in implementing network strategies. Perceived barriers included interprofessional collaboration, professional knowledge, self-efficacy, and financial structures.

**Conclusions:**

Strategies to involve the social network of people post-stroke are not fully implemented. Although identifying members of a social network should begin during inpatient rehabilitation, the main part of actively engaging the network will have to take place when the people post-stroke return home. Implementing social network strategies requires a systematic process focusing on collaboration, knowledge, attitude, and skill development.

**Supplementary Information:**

The online version contains supplementary material available at 10.1186/s12875-022-01897-3.

## Background

Stroke can lead to a loss of independence; it also places a huge financial burden on countries and is a major cause of mortality [1]. People who have strokes tend to be less able to engage in the activities they valued doing before, such as their social, family, or leisure activities [2–4]. Engagement in valued activities is associated with higher levels of well-being and quality of life [5–7]. However, in the long run, people post-stroke find it difficult to maintain the skills they acquired during rehabilitation [8, 9]. Months to years after the stroke, the majority stopped participating in their most valued activities [3, 10, 11] and are at risk to become homebound and sedentary. Social relationships are more difficult to sustain [12, 13] which may result in social isolation [14–16].

To ensure that people post-stroke can maintain their activities and social relationships in the long run, it is important that rehabilitation professionals prepare people post-stroke for when they return home and give them the necessary support [9, 17]. Several authors have suggested that members of the broad social network (extended family members, friends, neighbours, or colleagues) should play an active role in the early phase of rehabilitation and that they should be taught how to provide adequate ‘activity support’ [18–22]. Members of the network should be informed about the importance of activity (re)engagement post-stroke and learn support strategies [18, 19, 22, 23]. This can improve activity re-engagement as a paramount rehabilitation outcome [18–20, 22], and ease the burden on the primary caregiver [24].

It is not clear when and how the broad social network can be best equipped to provide adequate activity support. During inpatient rehabilitation, most patients and caregivers are not aware of the risk of loss of engagement in valued activities and do not want to burden or engage their social network [25]. Also, because of rising healthcare costs, the length and intensity of inpatient rehabilitation trajectories is downsized. Care delivery is shifting from specialized inpatient care to home-based services and informal caregiving [26–28]. So besides knowing how the social network can be equipped to provide support, this transition asks for specific implementation strategies that support integrated rehabilitation and community care. Contextual determinants need to be identified and implementation strategies need to be developed to support the implementation of social network strategies. The CFIR (Consolidated Framework for Implementation Research) can help in this regard [29] because its comprehensive and multifaceted nature matches the complexities of transformative interventions [30].

## Methods

### Aim

The present study aimed to: (1) identify, based on the experiences and insights of stroke professionals, strategies that appear to be effective in optimising the ability of the broad social network members to support the activity (re)engagement of people post-stroke; (2) understand when these strategies are best implemented; and (3) recognize the factors that hinder or facilitate such implementation.

### Study design

The present study employed a constructionist epistemology. The constructionist epistemology theorizes that reality is socially constructed, which implies a unique view of the world for each person in line with someone’s perception and description of themselves and their reality [31, 32]. We used focus group meetings to construct professionals’ perspectives about strategies that could establish a supportive social network and factors that may influence implementation. We anticipated that focus group meetings could maximise the exploration of different perspectives and would therefore lead to more in-depth insights than individual interviews.

### Setting

This study was conducted with medical and allied health professionals from hospitals, rehabilitation centers and community care centers in the Netherlands.

### Participants recruitment

Convenience sampling was used. Medical and allied health professionals known in the research group network, were invited to participate if they had at least 2 years of experience working with people post-stroke. One of the authors (SJ) contacted the individuals and informed them of the study aims and procedure, guaranteed anonymity and confidentiality regarding data management and possible publication, and used snowball sampling to gather other recruits.

### Data collection

The data were collected during the focus group meetings which were held at the HAN University of Applied Sciences, a neutral and unfamiliar location for most of the participants.

The participants were asked to give written informed consent to the audiotaping of the meetings. An interview guide, developed by the research group during three consensus meetings, was used. The questions were nondirective and open-ended (Appendix 1) [33]. The research group consisted of five experts from different allied health professions. They were all trained as clinicians and academics and had extensive experience of working with stroke survivors in a clinical setting.

Because the participants were unfamiliar with the location and each other, it was important to ensure they felt comfortable. They were asked to provide examples of instances in which social network members facilitated or hindered the activity (re)engagement of people post-stroke. Next, they were asked to consider the study’s key questions, which related to strategies that establish a supportive social network during inpatient and community rehabilitation and to factors that influence the implementation of these strategies.

The meetings were moderated by a researcher (DV) who had experience in moderating focus group discussions. When appropriate, she asked the participants to elaborate on their answers. Another researcher (SJ) asked additional questions when required. Once all the information had been collected a research assistant (KB) provided a brief summary. Finally, DV asked whether anything had been missed.

### Data analysis

The audiotape recordings were transcribed verbatim. Each participant was assigned a person-specific ID number on the transcripts to assure anonymity. To identify key issues and patterns, DV and KB carried out a conventional content analysis [34] using Atlas.ti version 7.5.2. This type of analysis is used to extract information without the imposition of preconceived categories or theories [34]. The researchers DV and KB independently analyzed the data through a line-by-line review of the transcripts and identified the text units that related to the influence of the social network, the strategies that could establish a supportive social network and factors that might negatively or positively impact their implementation. The highlighted text units were then coded using open coding [34]. Codes referring to the same key issue were grouped, allowing distinct categories to emerge. A discussion between researchers (KB, DV, SJ) was held to establish a consensus on codes and categories, and these were presented to the research group who then came to a consensus on overarching themes. Data relating to the factors that might influence the implementation of strategies were categorised within the domains of the CFIR by DV and ES [29].

## Results

Thirty-four professionals were approached via e-mail or phone. Eighteen professionals (with an average of fifteen years of experience with stroke care) agreed to participate. A workable and effective focus group generally encompasses up to ten participants [35]. Two focus group meetings (of nine participants each) were planned. Participants were allocated to one of the two groups based on their date and time preferences. When no preference was reported, the participant was allocated to the less heterogeneous group. This ensured the maximum possible heterogeneity within the groups and homogeneity between the groups. Table 1 shows the characteristics of the participants.

**Table 1:** Demographic data and characteristics of focus group participants

		Group 1 (*n* = 9)	Group 2 (*n* = 9)
Age (years)	*Average*	40.2	44.8
	*Range (min-max)*	26–60	31–58
Years in practice	*Average*	14.4	16.5
	*Range (min-max)*	2–30	2–30
Gender (n)	Male	2 (22%)	1 (11%)
Discipline (n)	Social worker	1 (11%)	0 (0%)
	Nurse	2 (22%)	2 (22%)
	Physical therapist	4 (44%)	3 (33%)
	Speech therapist	1 (11%)	1 (11%)
	Occupational therapist	1 (11%)	3 (33%)
Setting (n)	Community care	2 (22%)	1 (11%)
	Rehabilitation center + community care	0 (0%)	2 (22%)
	Rehabilitation center	1 (11%)	5 (56%)
	Hospital	6 (67%)	1 (11%)

The participants mentioned a variety of possibly effective strategies, some of which were already in use and others that could be implemented or developed. Then they discussed when, how and under what circumstances the strategies could be introduced. The CFIR constructs were used to summarise the various factors that might influence implementation.

The mentioned strategies to equip the social network to support people post-stroke relate to: identifying and expanding the network, informing the network, and activating network support (Table 2).

**Table 2:** Mentioned social network strategies

Strategies in use	Strategies that could be implemented or developed	Barriers (−) and facilitators (+) implementing strategies
***Theme 1: Strategies to identify and expand the social network***		
**To identify the network** **• Using a Ecomap (diagram of social and personal relationships) to visualize all members and discuss their potential.** **• Using the Mantelscan [in Dutch] (informal care-scan) to visualize the structure, organization and risk factors of a person’s network.** **• As an element of a ‘regular intake’.** **To expand the network** **• Encouraging people to reactivate former relationships.** **• Encouraging people to undertake (new) activities with others.**	To identify the network **•** Using a questionnaire to screen the network for crucial signs of vulnerability before discharge. **•** Using a card system to pass on signals regarding the vulnerability of a social network.	˗ Participants felt insecure about mapping the social network. ˗ Participants (inpatient and community settings) experience not enough time to identify the social network. + Participants from inpatient settings can screen the network for the most crucial signs of vulnerability.
***Theme 2: Strategies to inform the network***		
**• Organizing individual and group education sessions to provide information about stroke and its consequences.** **• Organizing follow-up care to stay in contact and inform the network after discharge.** **• Using an “I am changed document” consisting of information in layman’s terms regarding a person’s abilities and difficulties.** **• Using communication apps to share photos and information with network members.**	**•** Developing and using e-health courses tailored to the specific information need(s) of the person post-stroke and their network.	+ Social network members are interested in education after discharge. ˗ Contact with people post-stroke and their network can be easily lost. ˗ General practitioners and practice nurses do not (sufficiently) focus on the network. ˗ Privacy legislation hinders sharing patient-specific information with members of the network. + The availability of technological tools.
***Theme 3: Strategies to activate network support***		
**• Using exercise apps to facilitate network members in guiding the person post-stroke in performing their exercises.** **• Inviting social network members to practice with the person post-stroke and inform them, show them, and let them experience what the person is capable of.**	**•** Providing a buddy who can give support through each phase after stroke, gather and process information and inform the rest of the network if necessary.	˗ Participants from inpatient settings have limited options to work with members of the network. ˗ Privacy legislation hinders sharing patient-specific information with members of the network. ˗ People post-stroke are unaware of the needed support and therefore do not (want to) include network members. ˗ The division of tasks between professionals regarding working with the network is unclear.

### Strategies to identify and expand the social network

The participants agreed that it was first necessary to identify the members and characteristics of the network. In the experience of one participant (a social worker), using a ‘network map’ that visualizes the members and discussing what they can contribute, makes it easier for people post-stroke to ask for support. The participants believed this could prevent burden on the primary caregiver(s) and should be implemented as soon as possible after the individual experienced the stroke.

*“Often, people do not see the skills that are available within their network. However, when you look at it [i.e., the network map] together, it makes someone less hesitant about asking family members, friends or others for help.”* (Social worker, rehabilitation centere)

Some participants pointed out that visualising a network clarifies whether measures should be taken to expand the network. For instance by encouraging people to reactivate former relationships or undertake (new) activities with others.

Although one of the participating social workers assured the group that no specific knowledge or experience was required to map a social network, several participants felt insecure about how to do it themselves. Some of the participants from both inpatient and community rehabilitation said they did not have the time.

*“Dutch health insurance actually requires community nurses to map the network as part of the intake procedure. However, during the first visit, I talk about the client’s concerns, which usually takes a lot of time. So I need to set priorities and focus on their initial request for help.”* (Community nurse)

The participants who worked in inpatient settings said that they could screen the network for crucial signs of vulnerability before discharge, for instance by asking the person post-stroke or a close loved one to fill out a questionnaire about the network’s characteristics. Based on these outcomes, community professionals could then decide whether actions concerning the social network are necessary and if so, how they should be prioritised. The participants working in community care welcomed this suggestion. They felt that currently, network information gathered during inpatient care was often not shared, which made it difficult for them to know whether the capacity of the social network was sufficient for it to support activity (re)engagement post-stroke.

### Strategies to inform the network

Although most of the participants were not used to working with a broad network during rehabilitation, they acknowledged the importance of informing the network and thought this should start in the early phases of rehabilitation. Several participants stated that they regularly organised group and individual education sessions in which they provided information about stroke and its situation-specific consequences. During these sessions, people post-stroke were encouraged to play an active role by sharing their experiences and support needs. Although the participants said that network members expressed little interest in pre-discharge sessions, the ones that took in the home environment were often well attended and well received.

The participants had observed that the need for information tended to increase over time amongst network members.

*“During rehabilitation, clients and network members are given a lot of information and you might think they had enough. However, after inpatient rehabilitation, they experience new problems at home [so additional information is needed]. Group training over several sessions addresses this issue.”* (Speech therapist, hospital)

The participants mentioned several professionals who could play a role in informing the network after discharge, such as community or stroke nurses. Because people post-stroke and their networks are subject to change over time, they also expressed the need for long-term follow-up and support. Organising follow-up care seemed especially important because contact with people post-stroke and their network can be easily lost. The participants thought that general practitioners and practice nurses should extend their reach and focus on the network of people post-stroke.

*“The general practitioner or practice nurse could explain the importance of having a [strong] network and ensure that it is able to cope and provide support. In the end, a supportive network will also save them time.”* (Community nurse)

Participants suggested that in more severe cases, a personal case-manager is warranted. This role was often played by community social workers, who coached people post-stroke on how to deal with day-to-day situations.

*“I go wherever it is necessary to go. If a client has run away angry from their children’s day care center, I go there and explain […] so the people there will understand.”* (Community social worker/case manager)

The participants mentioned that they regularly use technologies, such as communication apps, to inform network members. These could be used to share photos and information. They suggested that e-health courses tailored to the specific information need(s) of the person post-stroke and their network could be developed.

### Strategies to activate network support

The participants stated that rehabilitation professionals should not only inform the social network but also show them how to facilitate activity (re)engagement and encourage them to play an active role.

*“... just inviting them to practise with the person post-stroke, letting them experience what the person is still capable of, familiarizing them with different ways of communicating, showing them how to swallow or stand, in other words, actively joining in rather than just watching or listening.”* (Physical therapist, rehabilitation center)

The participants who work in inpatient rehabilitation bemoaned the fact that they had limited options to actually work with the broad network. Also, privacy legislation hindered the sharing of patient-specific information beyond the person post-stroke and their primary caregiver. The participants had found that people post-stroke seemed to be unaware of the support they might need after discharge. As a result, they may not understand why their network members need to play an active role in the early phases of rehabilitation.

*“Before discharge, people often expect everything to turn out all right in time. Asking others for support is like admitting you may not fully recover from stroke”* (Occupational therapist, hospital)

Because the participants encountered barriers when working with the broad network during inpatient rehabilitation, they suggested that working with the social network should take place mainly at home. This could involve providing a personal ‘buddy’ during the early stages of the rehabilitation trajectory. This buddy could support the person post-stroke through each phase, gather and process information and inform the rest of the network if necessary. The participants suggested that the buddy could be a network member one step removed from the immediate family, a volunteer, or a person post-stroke peer.

In general, the division of tasks between different professionals with regard to working with the network was seen as unclear. Also, the participants felt hindered in actively engaging the social network because hours spent working with them are hardly or not financed at all.

### Implementing social network strategies

**Fig. 1:**
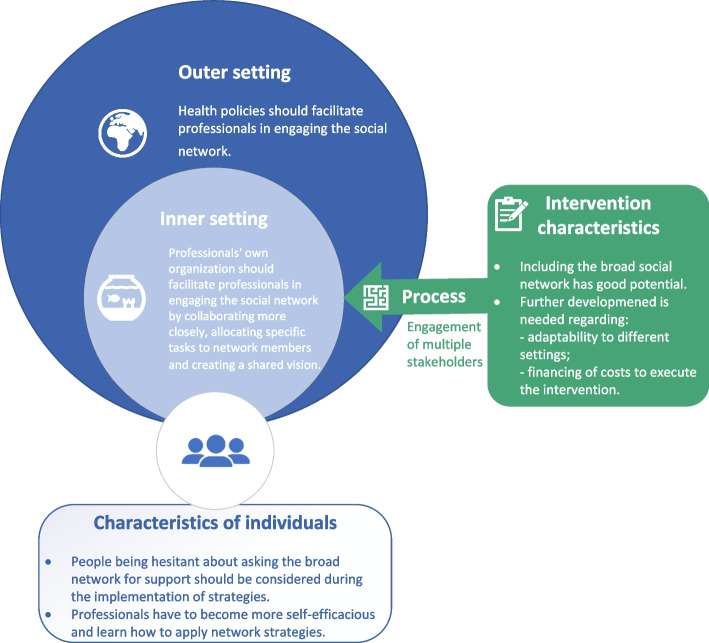
Factors influencing the implementation of network strategies per CFIR domain. Adapted from Khan [36].

Several factors that might facilitate or hinder the implementation of network strategies were mentioned. They fit well into the following CFIR domains: (1) the intervention; (2) the outer setting; (3) the inner setting; (4) the individuals; and (5) the process (Fig. 1) [29]. Our study shows that, concerning the first domain, participants perceived that including the broad social network has good potential. Further development will still be needed including adaptability to different settings and financing of costs to execute the strategies. The outer setting (health policies) and the inner setting (the professionals’ own organisations) should facilitate professionals in engaging the social network. Further action is also needed with respect to the fourth domain because people post-stroke and their primary caregivers are often hesitant about asking their broad network for support during rehabilitation. Finally, the professionals involved have to become more self-efficacious and learn how to apply network strategies. Implementing them requires the engagement of multiple stakeholders because barriers exist at multiple levels.

## Discussion

The present study examined the attitudes of stroke rehabilitation professionals towards strategies to engage the broad social network in enhancing activity resumption of people post-stroke. The participants suggested several strategies to identify (the characteristics of) the network, to inform social network members and to actively engage them. As has been noted, some of these strategies were already in used, while others could be implemented or developed. Several factors that seemed to facilitate or hinder the implementation of network strategies were mentioned. Our and previous studies shows that the restricted financing [28, 37] and the hesitation of people to involve the broad social network during rehabilitation [25], hinders the implementation of social network strategies. According to Jellema et al., people are hesitant because they expect the person post-stroke to resume activities after discharge without help from others [25]. A study shows that, when family members are actually included, people post-stroke and their family members are positive about the process and results of rehabilitation [38]. Implementation of social network strategies should therefore include the participation of people post-stroke and network members. Also, the implementation of strategies to improve the quality of care calls for professionals and their organisations to collaborate and communicate more closely, allocate specific tasks to network members, and create a shared vision and goals [39, 40]. According to MacInnes et al., co-designing and implementing integrated care can bring professionals together, strengthen relationships and help to understand each other’s roles and responsibilities [40]. Although implementing network strategies will entail extra costs, it will save costs in the long run. After all, if people post-stroke can resume their lives and continue to engage in activities they value, caregivers will be less burdened and unnecessary secondary costs may be avoided. Implementing social network strategies requires a systematic stakeholder-shared process of change that focuses on knowledge, greater awareness, a shift in attitudes, and skills development. Engagement of social members during routine stroke inpatient and community rehabilitation will then make activity re-engagement post-stroke more likely.

### Strengths and limitations

Because the study participants (including the focus group moderator) were familiar with different aspects of post-stroke rehabilitation, a range of perspectives could be collected. The use of a focus groups can give rise to opinion contamination. However, in the present case, the participants reported their thoughts, without any suspicion that their expertise was being questioned. While a variety of professional perspectives were gathered, those of a psychologist or general practitioner were not. However, since the participants worked as part of multidisciplinary stroke teams that included psychologist and general practitioner, we concluded that their contributions were sufficiently valuable to continue with the study. In further research, the opinions of psychologists and general practitioners would certainly be worth seeking out, especially when building a collective vision of social network strategies and making these strategies part of coordinated, routine, long-term integrated stroke care.

## Supplementary Information

**Additional file 1.**

## Data Availability

The datasets used and/or analysed during the current study are available from the corresponding author on reasonable request.
