# Supplementary material for: Enhancing the role of the social network in activity (re)engagement post-stroke: a focus group study with rehabilitation professionals
Source: BMC Prim Care. 2022 Nov 17;23:285. doi: 10.1186/s12875-022-01897-3 (PMC9673324; doi:10.1186/s12875-022-01897-3)
Supplement: Supplementary file 1 — Additional file 1. [file 12875_2022_1897_MOESM1_ESM.docx]

# Appendix 1

**Focus group interview guide**

*Establishing a social network that supports activity (re)engagement of people post-stroke*

1. **Introduction (max. 25 minutes)**

Welcome

Participants are welcomed with coffee and tea. Chat informally to make them comfortable and create a relaxed ambiance. Participants are randomly seated behind tables (set in a square position).

Introductions

- Introduce the focus group leader (name, organization and job role), second researcher (name, organization and job role) and research assistant (name, organization and job role).
- Ask permission to tape record the meeting. Information will be used anonymously.
- Ask participants to name their occupation, work setting, working years and years of experience treating clients in (home-based) stroke rehabilitation.
- Propose communicating on a first-name basis.

Introduction to the focus group method

- Duration: 2 hours (with a break halfway for drinks).
- Goal: Identify strategies that can stimulate the broad social network to support the activity (re)engagement of people post-stroke and discuss which factors influence whether you can implement these strategies in daily practice.
- Clarify the roles of the focus group leader, second researcher and research assistant.
- Explain the focus group technique and encourage people to share their visions (e.g., no right or wrong answers, opinions may vary).

1. **Starting the focus group discussion**

- Introduce the subject of discussion by defining ‘broad social network’ and ‘activity (re)engagement.’
- Explain the purposes of the focus group: (1) to identify strategies that can stimulate the broad social network to support the activity (re)engagement of people post-stroke, and (2) to discuss which factors influence whether you can implement these strategies in daily practice.
- Ask for verbal informed consent to audiotape the interviews, including how data will be handled (data saved and ID number assigned to each participant).

*Part A: identify network factors that hinder or facilitate activity resumption
Participants’ perceptions and experiences regarding the influence of the broad social network on the activity (re)engagement of people post-stroke.*

**Goal**: Collect different experiences and examples in which the broad social network facilitated or hindered activity (re)engagement.

**Assignment 1**: “Try to remember a case in which the broad social network hindered activity (re)engagement. Write down which of the broad social network’s actions or characteristics were hindering.”.

**Questions** to start the conversation: “Who wants to share their experience?”. **Follow-up questions**: ”Where did this take place (community care/institution)?”, ”What happened?”, ”What was the consequence?”, ”What do you think caused this behavior?”, ”Is there anyone (participant) who recognizes this situation?”.

**Assignment 2**: “Try to remember a case in which the broad social network facilitated activity (re)engagement. Write down which of the broad social network’s actions were stimulating.”.

**Questions** to start the conversation: ”Who wants to share their experience?”. **Follow-up questions**: ”Where did this take place (community care/institution)?”, ”What happened?”, ”What was the consequence?”, ”What do you think caused this behavior?”, “Is there anyone (participant) who recognizes this situation?”.

*Researcher presents results from prior research concerning the influence of the broad social network on activity (re)engagement of people post-stroke.*

*Part B: key question 1*

*Strategies that can transform an ‘obstructing’ broad social network into a ‘facilitating’ broad social network*

**Goal:** Identify strategies that can stimulate the broad social network to support the activity (re)engagement of people post-stroke.

**Assignment**: “Think about strategies that can help transform an ‘obstructing’ broad social network (mentioned during warm up) into a ‘facilitating’ broad social network (mentioned during warm up). Write down these strategies.”.

**Questions** to start the conversation: “Who wants to describe one of the strategies?” **Follow-up questions**: “What does this strategy include?”, “Where can this strategy take place (community care/institution)?”. **Follow-up question: “**Do you know examples of (standardized) methods to create a supportive broad social network?”, What does it include?”, “Who can use it?”, “Where?”, “How?”.

*Part C: key question 2*

*Implementing strategies*

**Goal**: Collect different opinions about possible/experienced facilitators and barriers concerning the implementation of strategies aimed at creating a broad social network that supports the activity (re)engagement of people post-stroke.

**Topic 1**: Role of professionals implementing/executing the strategies and/or methods (mentioned in part B).

**Questions**: “Which professionals should implement/execute the strategies/methods?”, “Why?”, “When (during which phase of rehabilitation/in community or institutionalized care)?”.

**Topic 2**: Preconditions for being able to implement/execute the strategies

**Questions**: ”What do professionals/you need to be able to implement/execute the strategies/methods?”, “Who should be engaged?”, “How should they collaborate?”,

**Topic 3**: Factors influencing the implementation of strategies/methods

**Questions: “**What are barriers?”, “What are facilitators?”

During the focus group

The focus group leader encourages people who seem to be reserved to express their opinions by individually asking questions about their experiences or opinions.

When saturation seems to be reached, the focus group leader evaluates whether all questions have been answered. If necessary, participants will be asked to further elaborate on specific topics from the interview guide.

1. **Ending the focus group**

Before ending the focus group meeting, the research assistant provides a brief summary of the discussion for verification purposes. If participants agree that the most important issues concerning the key topics have been mentioned, the meeting will end.

*Official ending:*

- Thank the participants for participating and contributing.
- Inform them about future steps: analyzing data, reporting back to interested participants and publishing findings (in a scientific journal).
